# Percutaneous exposure incidents in nurses: Knowledge, practice and exposure to hepatitis B infection: Percutaneous exposure incidents in nurses

**Published:** 2011-03-01

**Authors:** Navid Mohammadi, Abbas Allami, Rasoul Malek Mohamadi

**Affiliations:** 1 Faculty of Medicine, Tehran University of Medical Sciences, Tehran, IR Iran; 2 Faculty of Medicine, Qazvin University of Medical Sciences, Qazvin, IR Iran; 3 Faculty of Medicine, Shahid Beheshti University of Medical Sciences, Tehran, IR Iran

**Keywords:** Knowledge, Practice, Exposure, Hospital, Needlestick

## Abstract

**Background:**

Nurses are at risk of percutaneous exposure incidents (PEIs), which may lead to serious or even fatal blood-borne infections.

**Objectives:**

To determine the prevalence of PEIs in the last year, among nurses and to assess their knowledge about and frequency of safe method of practice in exposure to blood-borne pathogens (especially, to HBV).

**Materials and Methods:**

A cross-sectional study in 2008 was conducted on 138 nurses working in general surgery and obstetrics/gynecology services of Qazvin University of Medical Sciences, Qazvin, Central Iran. A questionnaire for assessment of risk factors for contracting HBV infection was completed by nurses.

**Results:**

Overall, the prevalence of needle stick injury (NSI) and direct exposure to body fluids were 52.9% (95% CI: 44.5%-61.3%) and 65.4% (95% CI: 57.4% - 73.8%), respectively. There was no statistically significant difference between the two studied centers in terms of sharp injuries; however, the rate of repeated NSI (number per each year ≥3) and mucocutaneous exposures were significantly higher in the general surgery ward. The overall coverage of vaccination in the two studied centers was 96.3%, but the rate of accurate answers to many questions pertaining to knowledge and practice were less than 50%.

**Conclusions:**

Nurses are still at significant risk for developing NSI and mucocutaneous exposure. Continuous educational programs (especially by highlighting the seriousness of the problem) are necessary for improving this situation because inadequate education might increase unsafely practice.

## Background

Percutaneous exposure incidents (PEIs) (needle stick injuries, sharp injuries, as well as splashes leading to exposure of the skin or mucosa to blood) constitute a major occupational hazard for health care workers [1][2][3]. Nurses are facing the threat of PEIs with the consequent risk of contracting blood-borne infections caused by pathogens such as hepatitis B and C viruses [4]. The results of the assessment of hygiene practices of health care workers and compliance with the recommended instructions among staff of 30 hospitals in Shiraz, southern Iran, showed that physicians and nurses were less compliant with personal hygiene practices than cleaners [5]. Although occupational HIV and hepatitis seroconversion is relatively rare, the risks and associated costs of a blood exposure are serious and real. These costs include initial and follow-up treatment of the exposed health care personnel [6][7], fear and anxiety about the possible consequences of an exposure [8], drug toxicities and absence from work. Previous studies on needle stick injuries (NSI) in Iran were mainly focused on the prevalence of the injuries [9][10]. However, recent studies focused on the factors that may be associated with this problem which include individual factors (e.g., previous history of HBV vaccination, level of knowledge of blood-borne diseases and universal precautions, practice by complying with universal precautions and perception concerning the consequences of NSI) among nurses [11][12]. These data are essential for targeting and evaluating interventions at local and national levels. Periodic surveys can be used to monitor improvement in measures [13].

## Objectives

We therefore, conducted this study to determine the prevalence of PEIs in the last year among nurses and to assess their knowledge about and frequency of safe method of practice in exposure to blood-borne pathogens (especially, to HBV) in two educational hospitals-centers for general surgery and obstetrics/gynecology.

## Materials and Methods

In an analytical cross-sectional study conducted in 2008, 150 nurses working in two educational hospitals-centers for general surgery and obstetrics/gynecology-were asked about their personal experiences in regard to PEI during the past year. A self-administered questionnaire consisting of 27 closed questions was used. Nurses were asked to complete the questionnaire and returned it within four days. Several questions were asked about their knowledge of blood-borne diseases and universal precautions, their experience in handling needles and the prevalence of NSI and other mucocutaneous exposures during the past year. The questionnaire had 12 questions for assessment of knowledge and 15 questions for practice of universal precautions (according to the guidelines) [14].

We distributed questionnaires to all 150 nurses working in the two educational hospitals. Data analysis was done with SPSS® ver 13 (SPSS Inc., Chicago, Ill, USA). Quantitative variables were presented as mean ± SEM (standard error of mean). x(2) test or Fisher's exact test when appropriate were used to compare qualitative variables (factors associated with NSI) and to find out the difference between the two studied groups (surgery and obstetrics/gynecology nurses). A p < 0.05 was considered statistically significant.

## Results

Of 150 questionnaires distributed, 138 were completed and returned translating to a response rate of 92%. Of the 138 completed questionnaires, 87 belonged to general surgery and 51 to obstetrics/gynecology wards. In total, 73 (52.9%; 95% CI: 44.5%-61.3%) of the 138 respondents reported NSI at least once in the past year (Table 1). The prevalence of episodes of NSI was 63% (32 of 51; 95% CI: 49%-76%) in the obstetrics/gynecology ward and 47% (41 of 87; 36%-58%) in general surgery ward-not statistically significant (Table 2).

**Table 1:** Characteristics and answers to questions by the studied nurses

**Characteristics**	**Frequency** (%)		
	**Surgery**	**Obstetrics/Gynecology**	**Total**
**History of NSI episode**			
**Never**	41 (47)	18 (35)	59 (42.8)
**Once**	18 (21)	25 (49)	43 (31.2)
**Twice**	12 (14)	7 (14)	19 (13.8)
**More**	11 (13)	0 (0)	11 (7.9)
**Did not respond**	5 (6)	1 (2)	6 (4.3)
**Mucocutaneous exposure**			
**Yes**	75 (86)	20 (39)	95 (68.8)
**No**	12 (14)	31 (61)	43 (31.2)
**Immunization status**			
**Complete **(3 doses)	82 (94)	51 (100)	133 (96.4)
**Not complete**	5 (6)	0 (0)	5 (3.6)
**Post-HBV vaccination titration**			
**Yes**	52 (60)	51 (100)	103 (74.6)
**No**	35 (40)	0 (0)	35 (25.4)
**Conducted antibody titration after NSI **(% of total NSI)			
**Yes**	14 (16)	8 (16)	22 (15.9)
**No**	73 (84)	43 (84)	116 (84.1)
**Use of protective equipment**			
**Yes**	67 (94)	45 (98)	112 (95.7)
**No**	4 (6)	1 (2)	5 (4.3)
**Consumer type gloves**			
**Nylon**	2 (3)	2 (4)	4 (3.5)
**Latex**	28 (42)	10 (22)	38 (33.7)
**Nylon + Latex**	37 (55)	34 (74)	71 (62.8)

**Table 2:** Differences in exposure, knowledge of universal precaution and self-reported behaviors after injury among the studied nurses

	**Surgery**	**OB/Gyn a**	**odds ratio** (Point estimate)	**95**%** CI of odds ratio**	
				**Lower limit**	**Upper limit**
**History of NSI episodes**					
**At least once**	41	32	0.84	0.62	1.05
**Never**	41	18			
**Approach to discard of needles**					
**Correct**	23	26	2.05	1.24	2.87
**Incorrect**	64	25			
**Action to NSI**					
**Correct**	58	20	0.55	0.32	0.78
**Incorrect**	29	31			
**Mucocutaneous exposure**					
**Yes**	75	20	3.18	1.73	4.62
**No**	12	31			
**Immunization status**					
**Complete**	82	51	0.94	0.89	0.99
**Not complete**	5	0			
**Post-HBV vaccination titration**					
**Yes**	52	51	0.61	0.50	0.71
**No**	35	0			
**Conducted antibody titration after NSI (**%** of total NSI)**					
**Yes**	14	8	1.06	0.71	1.42
**No**	73	43			
**Use of protective equipment**					
**Yes**	67	45	0.89	0.75	1.02
**No**	20	6			

^a^ Obstetrics/Gynecology

The incidence of repeated NSI (≥ 3 times a year) was significantly higher in general surgery service (13.4%) than in obstetrics/gynecology ward (0.0%) (OR: 1.55; 95% CI: 1.08-2.22). Furthermore, the overall prevalence of direct contact with body fluids was 65.4% (95% CI: 57.4% - 73.8%); it was significantly higher in general surgery ward (91.46%) than obstetrics/gynecology ward (39.21%) (OR: 2.20; 95% CI: 1.50-3.25). In our study, 81.15% of all studied nurses (77.01% in surgery vs 88.23% in obstetrics/gynecology) had used gloves during high-risk procedures. The overall coverage of vaccination in the two studied hospitals was 96.3%. Post-vaccination test after 1-2 months of the vaccine series were performed in 74.6% nurses of whom 100% of nurses working in obstetrics/gynecology and 89.9% of those in surgery wards had developed protective antibody (anti-HBsAg) titers. Among 96 vaccinated nurses who rechecked their antibody levels, only 2% gave correct answers to questions about the best approach to NSI and exposure to patients' body fluids.

The correct response rate to the questions about history taking before aggressive procedures, wearing gloves during sampling from a patient, approach to a blood spot on a white coat, shedding patients' blood on the nurse body, and approach to discard of needles and cutter instruments was 57.1%, 95.7%, 18.1%, 57%, and 37.7%, respectively. For the latter item, there was a statistically significant difference between the two studied hospitals (51% for obstetrics/gynecology and 26.9% for the surgery ward; p<0.01). For the question on how to correctly act to a NSI by a patient with hepatitis B, the correct response rate was 56.5%, overall-39.2% for obstetrics/gynecology and 66.7% for surgery nurses (p<0.01). About 45.7% of responders believed that an infected nurse could continue their clinical duties with commitment to cautions and safety principles. We did not discover any HBV-infected nurse caused by occupational exposures.

## Discussion

In this study, 52.9% (73 of 132) respondents had sustained one or more NSIs during the past year. Although it is higher than that reported elsewhere [15][16][17][18], the true magnitude of NSI is difficult to assess in the absence of an integrated and careful monitoring system. In such a condition, information has not been gathered completely on the frequency of injuries among health care personnel working in hospital settings [15][19]. In our study, the incidence of repeated NSI was significantly higher in nurses of surgery ward. It's probably due to existence of more high-risk situations in the surgery settings (i.e., traumatic emergencies). In addition, many procedures in this field require needle handling such as venipunctures, parenteral injections to traumatic patients, and giving local anesthetics for performing invasive interventions [13]. On the other hand, another potential reason might be inadequate knowledge of surgical nurses. As previously mentioned, the rate of HBV vaccination coverage in surgery ward was higher than OB/GY. This might induced a false belief in them that they no longer need to protect themselves against NSI which ultimately results in a higher NSI [20]. Moreover, the rate of such injuries depends on the medical discipline; it seems that regarding this issue, the situation was better in obstetrics setting [21]. In accord with other studies, occupational exposure was not a major route for contracting HBV infection in nurses [22][23][24]. The incidence of HBV infection in health care personnel has declined steadily [13]. The decline in occupational HBV is largely due to widespread pre-exposure immunization and improved knowledge of health care personnel's about HBV risk factor [25][26][27][28][29]. This study found that most of the studied nurses (96.4%) had received the HBV vaccination. In another Iranian study conducted in 2000, the rate was 45.1% [30]. Comparison of these results shows that the rate of HBV vaccination coverage of health care workers in Iran has increased. Implementation of an in progress vaccination program against HBV in Iranian health care workers resulted in considerable reduction of use of high-titer immunoglobulin against HBV that is expensive (cost-effective strategy). Also, other studies demonstrated that this strategy decreases the anxiety of nurses after PEIs, and prevents the transmission of HBV after exposure in the majority of cases [31][32]. In spite of widespread HBV vaccination in nurses, susceptible ones are still at risk. Without post-exposure prophylaxis, probability of being infected by HBV for an exposed, susceptible health care worker is 6% to 30% (depends on the nature and frequency of exposure to blood or body fluids). In addition, PEIs expose health care worker to other blood-borne diseases [14]. In our study, knowledge and practice of nurses in many aspects were not adequate. In several subjects, the level of knowledge and accurate practice was low. Results of other studies in Iran are similar [24][33][34]. Several studies on the frequency of injury amongst nurses showed that systematic and continuing education correlated with a reduced frequency of NSI [35][36].

The two hospitals were different in some aspects that may be due to differences in activities of their infection control committees. Discipline in the obstetrics/gynecology ward is stricter than surgery services as the obstetrics/gynecology service had established and kept an updated record for all personnel and maintained the confidentiality of their records while ensuring that they are tested for HBsAg after any exposure and had received hepatitis B vaccination (and occasionally HBIG) when HBsAb titer was under 10 IU/dL. In the present study, the rate of mucocutaneous exposure with body fluids was incredibly high that is probably due to lack of caution during procedures (as a result of insufficient knowledge or shortage of necessary equipment). This finding is consistent with previous work in Iran [37] and other countries [2][38]. Findings of this study indicated that the prevalence of NSI among nurses is still high (despite widespread use of gloves). We assume that excessive handling of contaminated needles, high demands of patients for injections, and lack of safe needle and sharp containers enhance the risk of occupational transmission of blood-borne diseases such as hepatitis B. Although personal protective equipment (e.g., gloves, shields) provides a physical barrier for skin and mucous membranes from blood and other potentially infectious body fluids, they are easily penetrated by needles. In this study, some nurses (overall 20%) did not abide to the universal precautions such as wearing of gloves during high risk procedures. Several factors may account for this finding. Firstly, although use of gloves has increased in Iranian hospitals, cost of latex gloves continues to be a concern for managers and this may have been communicated to nurses-directly or indirectly-by managers. Secondly, gloves were not always found in the wards. Thirdly, it is possible that junior nurses were influenced by the behavior of their seniors who usually had not used personal protective equipment adequately. Another reason might be a perception that gloves interfere with nursing procedures or lead to complaints of patients so they prefer to avoid using gloves. In fact, since standard precautions focus on the use of physical barriers, we could not expect to see a significant impact on the prevention of sharp injuries and additional interventions (e.g., immediate and safe disposal of sharps into appropriate, puncture-proof sharps bins and using equipment designed with appropriate safety features) are needed [1]. This inadequate rate of use should be addressed in future training programs and revising local health care worker guidelines. In the present study, determination of antibody level had been performed for each case of NSI again, even in obstetrics/gynecology hospital (with a full coverage of vaccination against HBV and subsequent antibody determination). The centers for disease control and prevention (CDC) does not recommend routine post-exposure checking for vaccine responders despite HBsAg status of the source, thus this management may not be correct [39]. In fact, decrease of antibody concentrations under 10 IU/dL or even below detection levels is not considered as an indicator of loss of protection. Persisting protection against chronic hepatitis B carriage and clinical hepatitis B disease has been shown to last for long time [40][41][42][43][44]. The possibility of underreporting by nurses is a major limitation of our study. This limitation might be due to the recall problems to remember exactly the number of NSIs during past year. Also, nurses may have been embarrassed to admit their true number of needle stick and splash injuries as this may reflect bad practice on their part and so give a lower number. This study shows that nurses are still at significant risk for developing NSI and mucocutaneous exposure and therefore it is essential to pay more attention to their education in universities and continuous medical education (CME) programs because inadequate education might increase unsafe practice. Also, every hospital should have an established protocol to describe where and how their personnel should seek medical investigations and treatments after occupational exposure to blood or body fluids, including percutaneous injury. For finding appropriate ways to increase the nurses' information, more investigations are needed to study the reasons of this problem.
